# Flowers, Ferns, and Ward Decorations

**Published:** 1886-10-16

**Authors:** 


### FLOWERS, FERNS, AND WARD DECORATIONS.

Continued from page 28.—Communications for this column should be addressed, "Gardener," care of the Editor of THE HOSPITAL.

**The Charm and Beauty of Flowers.** ONE would find it difficult to discover in the whole horticultural category a bud or bloom without claim to charm and beauty of some kind or another, though I mind me of a plant known to me in my childhood, by the name of "The Devil," and present with me to this day by reason of its sombre colouring and obnoxious smell. It is a gruesome thing, well named, but withal first-cousin to the lords and ladies haunting the hedgerows in early spring, and a near relation of the pure white arum lily so common beside the banks of Father Nile. All flowers are beautiful, from therosy riches of Cachmire, which helped to set going the facile pen of Ireland's grandest poet, to the fair untinted edelweiss, ever raising its pale, enduring face to highest heaven from out the chill of perpetual Alpine snows, like a quiet handmaiden, born and reared in utmost desolation, beyond the reach of earth's tones, its restlessness, its rapture, untouched by its interest, its warmth, and its danger, living in absolute solitude in the unbroken solemnity of perfect peace, where no foot comes to break the calm of her infinite chastity, to disturb the crystalline purity of her doom, no whisper, no caress beyond a breath of icy coldness, alone, away from all living and breathing things, in endless patience, in faith immovable, waiting her release, always exalted, always strong, near to the countless stars, and nearest to God. In all ages flowers have been the theme of poet's song and painter's handiwork, used to express finest simile and happiest metaphor—in the beautiful figurative language of the East, in these our modern times, when, alas, they have furnished an outlet for the overwhelming absurdities of our latter-day rhymers. They were emblematical of life, of death, of honour, and of religion, since myrtle was bound upon the brows of heroes in those far-away ages, and roses decked the victims to be immolated upon the altars of old Hellenic gods.

**Christ and the Flowers.** Since then One walked the earth, many centuries ago, even He, who came to set a seal upon the bloody rites of ancient worship, whose horrors were but mocked by the presence of all that was rarest in the floral world—One whose loveliness and purity have been metaphorically set forth as "the Rose of Sharon and the Lily of the Valley"; beneath whose hallowed feet, those looking on at the time may have wondered to see no blossoms spring, as they trod the rugged barrenness of old-world Palestine, One from whose smileless life came forth sweet words, emphasising the simple beauty of some of His own most exquisite creations, when He contrasted the gaudiness of earthly splendour with the undecked lily of the field. It is supposed by some writers that the particular flower referred to by the Christ on this occasion was a little, low-growing, bulbous plant of pale yellow colour, not unlike a crocus in form, and known botanically as *Sterubergia Lutea*, and not, as is commonly believed, the well-known lily of the valley. Others have said that one of the numerous family of Asphodels was in our Lord's mind at the time; but none have been able to speak with unimpeachable authority.

(*To be continued.*)

